# Antimicrobial therapy in obesity: a multicentre cross-sectional study

**DOI:** 10.1093/jac/dkv189

**Published:** 2015-07-14

**Authors:** Esmita Charani, Myriam Gharbi, Gary Frost, Lydia Drumright, Alison Holmes

**Affiliations:** 1 National Institute of Health Research Health Protection Research Unit, Imperial College London, Hammersmith Campus, London W12 ONN, UK; 2 Department of Medicine, Imperial College London, Hammersmith Campus, London W12 0NN, UK; 3 Department of Medicine, University of Cambridge, Addenbrooke's Hospital, Hills Road, Cambridge CB2 0QQ, UK

## Abstract

**Objectives:**

Evidence indicates a relationship between obesity and infection. We assessed the prevalence of obesity in hospitalized patients and evaluated its impact on antimicrobial management.

**Methods:**

Three National Health Service hospitals in London in 2011–12 were included in a cross-sectional study. Data from all adult admissions units and medical and surgical wards were collected. Patient data were collected from the medication charts and nursing and medical notes. Antimicrobial therapy was defined as ‘complicated’ if the patient's therapy met two or more of the following criteria: (i) second- or third-line therapy according to local policy; (ii) intravenous therapy where an alternative oral therapy was appropriate; (iii) longer than the recommended duration of therapy as per local policy recommendations; (iv) repeated courses of therapy to treat the same infection; and (v) specialist advice on antimicrobial therapy provided by the medical microbiology or infectious diseases teams.

**Results:**

Of the 1014 patients included in this study, 22% (225) were obese, 69% (696) were normal/overweight and 9% (93) were underweight. Obese patients were significantly more likely to have more complicated antimicrobial therapy than normal/overweight and underweight patients (36% versus 19% and 23%, respectively, *P* = 0.002). After adjustment for hospital, age group, comorbidities and the type of infection, obese patients remained at significantly increased odds of receiving complicated antimicrobial therapy compared with normal/overweight patients (OR = 2.01, 95% CI 1.75–3.45).

**Conclusions:**

One in five hospitalized patients is obese. Compared with the underweight and normal/overweight, the antimicrobial management in the obese is significantly more complicated.

## Introduction

The global burden of obesity is reaching unprecedented levels. It is estimated that if the current trend in obesity continues, 3 in 4 of the US population and 7 in 10 of the UK population will be obese by 2030.^1^ The health implications are immense. Obesity is associated with significant health complications and morbidity, including diabetes, cancer and heart disease.^2^ Emerging data indicate a link between infection and obesity, with obese patients: (i) being at higher risk of infection, in particular healthcare-associated infection; and (ii) having poorer infection-related clinical outcomes.^3^ Obesity is a recognized, independent risk factor in surgical site infections following particular procedures (e.g. vascular and cardiothoracic surgery), where obesity significantly increases the risk of surgical site infections.^4^ Infections in critical care,^5^ cardiology,^6^ gynaecology^7^ and orthopaedics^8^ have also been reported as more prevalent among obese patients. Obese patients have also been identified as being at higher risk of respiratory tract infections^9,10^ and *Clostridium difficile*^11^ infections. These infections are a major health threat worldwide, with significant socio-economic impact on the health infrastructure. Despite the growing evidence implicating obesity as a major risk factor for healthcare-associated infections, little evidence exists as to the possible reasons for this association between obesity and infection.^12^

Obesity is most commonly defined using the standardized BMI equation: weight (kg) divided by height (m) squared. According to this WHO-ratified equation, a BMI of >30 kg/m^2^ is defined as obese and a BMI of >40 kg/m^2^ is defined as morbidly obese. Therefore, to understand the prevalence of obesity in populations, accurate information on weight and height of individuals is required. Despite not distinguishing between body fat and muscle, BMI is currently the most widely accepted and used scale to define obesity.

Adipose tissue can act as an endocrine organ and exert immunomodulatory effects on the body.^13^ Although the full effect of this immunomodulation in obesity is unknown, it is plausible that it may be a contributing factor to the observed outcomes of infection among obese people. One other factor contributing to the observed poorer infection outcomes among obese patients may be inadequate antimicrobial dosing due to both: (i) lack of weight-related dosing adjustment;^14^ and (ii) paucity of evidence on dosing due to lack of antimicrobial pharmacokinetic studies.^3,15^ The volume of distribution and renal clearance is increased in obese individuals and there are also changes in serum protein levels and hepatic metabolism.^15^ These physiological changes in obesity can alter the bioavailability of drugs and therefore have implications for drug dosing in obese individuals. In antimicrobial dosing, achieving the right concentrations at the site of infection is paramount both to ensure efficacious therapy and to prevent emergence of resistance by exposing pathogens to sub-therapeutic concentrations of antimicrobials. Despite this, there remains a significant gap in scientific literature on appropriate dosing of antimicrobials among obese patients for both treatment and surgical prophylaxis, with only limited and mainly anecdotal evidence from pharmacokinetic studies.^16^ Whilst there are overall population data on obesity and limited data on prevalence of obesity^17^ in hospitalized patients, there are no data on the relationship between obesity and antimicrobial therapy in practice. At the Imperial College Healthcare National Health Service (NHS) Hospitals Organisation we serve a densely populated urban community and have the largest bariatric centre in Europe. We report here on a cross-sectional study investigating the prevalence of obesity in hospitalized patients and the associations between obesity and antimicrobial therapy. Our aim was to measure the prevalence of obesity in hospitalized adult patients and evaluate the impact of obesity on infection and antimicrobial management in this population.

## Methods

### Study design and population

This was a cross-sectional study that included data collected in three hospitals of a multisite, 1500 bed NHS hospital organization in London. Data were collected in two cross sections between March 2011–September 2011 and July 2012–August 2012. Existing local antibiotic consumption data indicated that seasonal variability was not significant. Adult admissions units and medical and surgical wards on all three hospital sites were selected for the purpose of identifying the study population. The same wards were included in both episodes of data collection. The data for each patient were collected in a single day to provide a snapshot of patient episodes. All patients physically present on the ward at the time of data collection were eligible to be included in the study. Patients who were away from the ward for a procedure or an operation were excluded from the study, as were patients whose notes were unavailable at the time that the study investigators were on the ward. This was because patients who were physically absent from the ward could not be assessed for obesity, and patients whose notes were missing could not have their medical data included in the study.

### Ethics approval

Ethics approval to conduct this study was obtained from the Joint Research Compliance Office (JRCO) of Imperial College Healthcare NHS Hospital and Imperial College London. Formal approval from the National Research Ethics Service was not required for this research as the JRCO identified the work as hospital service evaluation and audit.

### Data collection

At the time of the study the hospitals did not have electronic medical records or prescribing systems and all data sources were paper records on the wards. A data collection form was designed and piloted prior to the study and all data were gathered using this single form. Patient medication charts and nursing and medical notes were screened to collect the data. Data were collected by author E. C. with assistance from specially trained researchers. Data entered into the final database were double checked for accuracy by one of the researchers.

To assess participants for obesity, recorded height and weight information was collected where it was available. Not all hospital wards had working scales and it was not possible to measure each patient's height and weight. Therefore, in addition to collecting the recorded height and weight from the medical records, a pre-validated figure rating scale (FRS) was used.^18^ The FRS used was a gender-specific, pictorial, BMI-based body size guide for men and for women. The scale had been validated in a study with 400 participants.^18^ To measure obesity using the FRS, each patient who was on the ward when the investigators were collecting data was independently assessed by at least one of the investigators and the result recorded. To assess inter-rater reliability of obesity using the FRS, wherever it was possible two investigators independently assessed the study participants using the scale.

Participant demographic data (e.g. age, gender and date of birth), medication history, reason for admission and comorbidity data were collected from the medical notes. Current medication, including antimicrobials (and the indication), for the admission episode was collected from the medication charts. For every participant, all courses of antimicrobial prescribing for an active infection were recorded. Antimicrobials administered for HIV and TB were excluded as patients would have been on these therapies for a long time. Antimicrobials prescribed for prophylaxis were also excluded from this study.

Patients were considered to have received a ‘complex antimicrobial therapy’ if they scored yes for two or more of the following criteria: (i) second- or third-line therapy according to local policy (second- and third-line therapy in the policy is reserved for more severe/complicated infections); (ii) intravenous therapy where an alternative oral therapy was appropriate; (iii) longer than the recommended duration of therapy as per local policy recommendations; (iv) repeated courses of therapy to treat the same infection; and (v) specialist advice on antimicrobial therapy provided by the medical microbiology or infectious diseases teams. The dose and frequency of all antimicrobial courses were recorded to assess whether any dosing adjustments were made based on weight for those antimicrobials that required it or whether any patients had their dose increased due to being overweight or obese.

### Data analysis

Demographic information, comorbidity information and infection-related outcomes of the study population were described and compared according to the patient weight category, using the χ^2^ test, Fisher's exact test and ANOVA tests where appropriate. To validate the accuracy of the FRS as a measure of obesity, the κ coefficient (a statistical measure of inter-rater agreement for categorical variables) was calculated for participants who had both a BMI (using recorded height and weight) and an FRS reading. Values of κ were interpreted using established thresholds.^19^

Univariate and stepwise multivariable logistic regression were used for the analysis of the association between patient weight category (using ‘normal/overweight’ as the reference group) and the composite outcome ‘complex antimicrobial therapy’. Exploratory univariate investigation of confounding and interaction were assessed for the following factors: patient weight category, study site, age, gender, ethnicity, and reason for admission, presence or not of comorbidities, i.e. diabetes and hypertension, number of specific comorbidities, number of current medications other than antibiotic and the type of infection. All variables with a *P* value <0.25 in an unadjusted univariate analysis were included in the multivariate logistic regression. A backward elimination approach was conducted to investigate the association between patient weight category and complicated antimicrobial therapy on a patient, adjusted for the confounding factors. Models were compared using a likelihood ratio test. The final model included covariates found to be significant in our regression model (*P* < 0.05) and was controlled for confounding factors identified in the literature, e.g. age. All analyses were conducted in STATA version 12 (StataCorp, College Station, TX, USA).

## Results

In each episode of data collection 34 wards were visited. These wards represent half of the available 1500 bed capacity across the hospitals included in this study. A total of 1338 (741 in 2011 and 597 in 2012) patients were admitted in a hospital bed on the day of data collection from the wards included in the study. Of these patients, 1056 (78%) were recruited as participants in the study. Due to incomplete data collection, 42 participants were excluded from the final analysis, so that the final number of participants in the study was 1014 (76%).

### Validation of the FRS

Height and weight were inconsistently recorded for the participants in their medical notes. This meant that BMI could only be calculated for 491 (48%) of the participants. The FRS was available for 100% of the participants. We found excellent agreement between BMI and FRS (κ = 0.823, SE = 0.036). As a result of this, the FRS was used to categorize participants as obese or non-obese.

### Prevalence of obesity and its impact on antimicrobial therapy

Of the patients assessed using the FRS (*n* = 1014), 22% (225) were obese, 9% (93) were underweight and 69% (696) were in the normal/overweight category. Women were significantly more likely than men to be obese (55% versus 45%, *P* < 0.001). Elderly people (>80 years) were significantly more likely than any other age group to be underweight than obese (38% versus 17%, *P* < 0.001) (Table 1).

**Table 1.** Univariate analysis of data by weight category

	Normal/overweight (*n* = 696)	Obese (*n* = 225)	Underweight (*n* = 93)	Total (*n* = 1014)^a^	*P*^b^
**Non-infection-related univariate analysis**					
Male (versus female), *n* (%)	386 (55.46)	101 (44.89)	33 (35.48)	520 (51.28)	<0.001
Age (years) (*n* = 1012), mean (SD)	65.57 (18.74)	65.25 (15.65)	70.97 (18.20)	65.99 (18.10)	0.0036
17–34	60 (8.63)	11 (4.91)	4 (4.30)	75 (7.41)	
35–64	228 (32.81)	79 (35.27)	23 (24.73)	330 (32.61)	
65–79	236 (33.96)	95 (42.41)	31 (33.33)	362 (35.77)	
80–100	171 (24.60)	39 (17.41)	35 (37.63)	245 (24.21)	<0.001
Ethnicity (*n* = 1003), *n* (%)					
white British	380 (55.15)	117 (52.70)	49 (53.26)	546 (54.44)	
white other	73 (10.60)	29 (13.06)	12 (13.04)	114 (11.37)	
black other/black Caribbean	58 (8.42)	21 (9.46)	4 (4.35)	83 (8.28)	
black African	39 (5.66)	11 (4.95)	6 (6.52)	56 (5.58)	
Indian/Pakistani/Bangladeshi	58 (8.42)	18 (8.11)	6 (6.52)	82 (8.18)	
other Asian	31 (4.50)	9 (4.05)	8 (8.70)	48 (4.79)	
other	50 (7.26)	17 (7.66)	7 (7.61)	74 (7.38)	0.824
Site, *n* (%)					
hospital 1	250 (35.92)	65 (28.89)	27 (29.03)	342 (33.73)	
hospital 2	211 (30.32)	86 (38.22)	24 (25.81)	321 (31.66)	
hospital 3	235 (33.76)	74 (32.89)	42 (45.16)	351 (34.62)	0.027
Accident and emergency admission, *n* (%)	364 (52.30)	101 (44.89)	52 (55.91)	517 (50.99)	
Infection, *n* (%)	184 (26.44)	59 (26.22)	28 (30.11)	271 (26.73)	
Planned surgery, *n* (%)	117 (16.81)	50 (22.22)	7 (7.53)	174 (17.16)	
Planned procedure, *n* (%)	26 (3.74)	11 (4.89)	3 (3.23)	40 (3.94)	
Other, *n* (%)	5 (0.72)	4 (1.78)	3 (3.23)	12 (1.18)	0.062
Comorbidities, *n* (%)					
diabetes	158 (22.70)	87 (38.67)	12 (12.90)	257 (25.35)	<0.001
hypertension	298 (42.82)	144 (64.00)	37 (39.78)	479 (47.24)	<0.001
Number of specific comorbidities, mean (SD)	3.52 (2.56)	4.40 (2.53)	4.19 (2.28)	3.78 (2.56)	<0.001
0	104 (14.94)	14 (6.22)	5 (5.38)	123 (12.13)	
1–3	260 (37.36)	67 (29.78)	33 (35.48)	360 (35.50)	
4–6	267 (38.36)	109 (48.44)	43 (46.24)	419 (41.32)	
≥7	65 (9.34)	35 (15.56)	12 (12.90)	112 (11.05)	<0.001
Number of all active medications (*n* = 994), *n* (%)					
0–4	155 (22.66)	29 (13.24)	11 (12.09)	195 (19.62)	
5–7	183 (26.75)	48 (21.92)	23 (25.27)	254 (25.55)	
8–10	170 (24.85)	60 (27.40)	28 (30.77)	258 (25.96)	
≥11	176 (25.73)	82 (37.44)	29 (31.87)	287 (28.87)	0.002
**Infection-related univariate analysis**					
Infection as reason for admission or acquired during stay, *n* (%)	331 (47.56)	115 (51.11)	46 (49.46)	492 (48.52)	0.639
**Patients who had an infection as reason for admission or who acquired subsequent infection in hospital (*n* = 492)**					
Infection by category, *n* (%)					
infection other	175 (52.87)	45 (39.13)	18 (39.13)	238 (48.37)	
hepatobiliary	23 (6.95)	9 (7.83)	0	32 (6.50)	
pneumonia	62 (18.73)	21 (18.26)	15 (32.61)	98 (19.92)	
surgical site infection	10 (3.02)	8 (6.96)	0	18 (3.66)	
skin and soft tissue	25 (7.55)	21 (18.26)	3 (6.52)	49 (9.96)	
urinary tract infection	36 (10.88)	11 (9.57)	10 (21.74)	57 (11.59)	<0.001
**Patients receiving antimicrobials for active infection (*n* = 421)**					
Complicated antimicrobial therapy	52 (18.51)	36 (36.00)	9 (22.50)	97 (23.04)	0.002
Number of antimicrobials/patient					
1	160 (56.94)	56 (56.00)	28 (70.00)	244 (57.96)	
2–5	121 (43.06)	44 (44.00)	12 (30.00)	177 (17.46)	0.265
Specialist input	29 (10.32)	17 (17.00)	1 (2.50)	47 (11.16)	0.036
Route of antimicrobials (*n* = 415)					
oral	145 (52.35)	52 (52.00)	27 (71.05)	224 (53.98)	
intravenous	128 (46.21)	48 (48.00)	11 (28.95)	187 (45.06)	
nasogastric	4 (1.44)	0	0	4 (0.96)	0.152

^a^Where *n* < 1014, due to missing data, the number (*n*) of participants is given in brackets next to the category in the table.

^b^Statistical significances are by χ^2^ test and Fisher's exact test.

Compared with the others, obese patients were significantly more likely to suffer from hypertension and diabetes. The number of comorbidities (e.g. hypertension, diabetes and cardiovascular diseases) was significantly greater in obese patients, with 16% of obese patients having more than seven comorbidities versus 9% in the normal/overweight category (*P* < 0.001) (Table 1).

Infection as a reason for admission was not significantly different across the weight groups. Planned surgery was a more likely reason for admission among obese patients. Skin and soft-tissue infections were significantly more prevalent among obese patients than the normal/overweight and underweight categories (18% versus 8% and 7%, *P* < 0.001). Surgical site infections were also more prevalent in obese patients compared with normal/overweight categories (7% versus 3%, *P* = 0.03). Obese patients were significantly more likely to have more complicated antimicrobial therapy than normal/overweight and underweight patients (36% versus 19% and 23%, respectively, *P* = 0.002). However, among the obese patients receiving systemic antimicrobials (421), we found no evidence of any dose adjustments made based on patient weight. Before adjusting for other factors the odds of obese patients having complicated antimicrobial therapy was greater than for normal/overweight patients (OR = 2.48, 95% CI 1.49–4.11); there was no significant difference between underweight and normal/overweight patients (OR = 1.28, 95% CI 0.57–2.85). After adjusting for hospital, age group, comorbidities (i.e. diabetes and hypertension) and the type of infection, obese patients remained at significantly increased odds of complicated antimicrobial therapy compared with normal/overweight patients (OR = 2.01, 95% CI 1.72–3.45) (Table 2), although this did not include any necessary dose adjustments for weight. No interactions between patient weight and other confounders were observed. No difference was observed in the odds of complicated antimicrobial therapy in underweight patients compared with normal/overweight patients.

**Table 2.** Multiple logistic regression examining the association between complicated antimicrobial therapy and weight after adjusting for potential confounders (*n* = 421)

Predictor	Unadjusted OR	95% CI	Crude *P*^a^	Adjusted OR	95% CI	Adjusted *P*^a^
Weight						
normal/overweight	reference			reference		
obese	2.48	1.49–4.11	<0.001	2.01	1.72–3.45	0.01
underweight	1.28	0.57–2.85	0.55	1.26	0.54–2.95	0.59
Age (years)						
17–34	reference			reference		
35–64	2.90	0.96–8.71	0.06	2.07	0.66–6.47	0.21
65–79	2.76	0.92–8.30	0.07	1.93	0.62–6.06	0.26
80–100	1.58	0.48–5.22	0.46	1.25	0.36–4.37	0.73
Gender						
male (versus female)	0.87	0.55–1.36	0.53			
Ethnicity						
white British	reference					
white other	1.64	0.80–3.38	0.18			
black other/Black Caribbean	1.17	0.45–3.09	0.75			
black African	1.24	4.67–3.27	0.67			
Indian/Pakistani/Bangladeshi	1.96	0.95–4.01	0.07			
other Asian	1.83	0.70–4.74	0.22			
other	0.70	0.26–1.91	0.49			
Site						
hospital 1	reference			reference		
hospital 2	1.88	1.04–3.40	0.04	1.98	1.06–3.71	0.03
hospital 3	0.97	0.52–1.83	0.94	0.93	0.48–1.81	0.83
Reason for admission						
Accident and emergency admission	reference					
infection	1.39	0.81–2.38	0.24			
planned surgery	1.51	0.77–2.96	0.23			
planned procedure	1.04	0.21–5.20	0.96			
other	1					
Diabetes						
yes (versus no)	1.26	0.76–2.08	0.37			
Hypertension						
yes (versus no)	1.46	0.93–2.31	0.10			
Number of specific comorbidities	1.02	0.94–1.11	0.59			
0	reference					
1–3	1.04	0.48–2.26	0.92			
4–6	1.69	0.81–3.54	0.16			
≥7	1.04	0.40–2.70	0.93			
Number of current medications						
0–4	reference					
5–7	1.25	0.53–2.98	0.611			
8–10	1.74	0.78–3.86	0.175			
≥11	2.03	0.95–4.36	0.067			
Type of infection						
infection other	reference			reference		
hepatobiliary	1.85	0.76–4.50	0.17	1.73	0.68–4.38	0.25
pneumonia	2.54	1.38–4.66	0.003	2.61	1.37–4.97	0.003
surgical site	3.10	1.13–8.51	0.03	2.75	0.94–8.02	0.07
skin and soft tissue	2.35	1.13–4.87	0.02	2.43	1.12–5.29	0.03
urinary tract	0.92	0.38–2.22	0.85	0.96	0.38–2.42	0.93

^a^Statistical significance is based on *P* < 0.05.

## Discussion

In this study, we found that obese patients were more likely to be receiving complicated antimicrobial therapy regimes in the hospital setting. More complicated antimicrobial therapy in the obese patient carries clinical and economic consequences, particularly as obesity is increasing in both developed and developing countries and antimicrobial resistance remains a potent public health threat.^1,11^ There have been several papers recently discussing the need to conduct further studies to investigate the impact of obesity on dosing of different antimicrobial agents.^3,14,16^ Obese patients remained at higher odds of receiving complicated antibiotic therapy after adjustment for confounding factors, such as age group, comorbidities (i.e. diabetes and hypertension) and the type of infection. Obese individuals were also more likely to be receiving intravenous antibiotics *and* be under specialist care *and* be receiving longer courses of therapy.

We found that no dose adjustments were made in the prescribing of antimicrobials for obese patients. The lack of antimicrobial dosing adjustments based on weight may mean that obese individuals who acquire infections are at higher risk of receiving sub-therapeutic antimicrobial levels.^19^ Sub-therapeutic dosing of antimicrobials can select for resistance. The threat that sub-therapeutic dosing of antimicrobials poses to emergence of drug resistance should not be underestimated, in particular in developing countries, where obesity is on the rise and access to effective and safe antimicrobials remains a problem, making the need to get therapy right the first time imperative.^20^

To make accurate recommendations about dosing adjustments in obesity, pharmacokinetic and pharmacodynamic studies are required for a large proportion of commonly used antibiotics, to measure the true association between body fat and antimicrobial levels. Until such studies are conducted, antimicrobial therapy in obesity will remain a grey area where clinical acumen, supplemented by anecdotal experience and limited data from small studies, will decide therapeutic outcomes.^21–25^ What is imperative is that dosing decisions are made based on drug- and patient-specific parameters. In the USA, attempts have been made to integrate antimicrobial dosing adjustments for obese patients. However, these initiatives have not been cohesively adopted and the practice of adjusting doses in obese patients remains erratic.^20^ In the absence of data from trials and clear guidelines on dosing adjustments in obesity for individual patients, there needs to be a more focused therapy for obese hospitalized patients who acquire infections. In this study, we did find antimicrobial therapies in the obese to be more complicated.

The prevalence of obesity in the hospitalized patients in this study reflects the increase in the prevalence of obesity in the UK, with one in five patients in this study being classified as obese. Half of the patients in this study were >65 years old, an age group more associated with increased weight loss and malnutrition;^26^ despite this, the prevalence of obesity remained high. In addition to the observed association between obesity and diabetes and hypertension, obese patients were also significantly more likely to have other comorbidities. This increase in disease burden, together with the associated medication burden, makes obese people more likely to be frequent users of healthcare.

In this study, skin and soft-tissue infections were more likely among obese patients, even when excluding surgical site infections. This association has been reported previously.^27,28^ We did not find a significant difference in infection as an admission reason across the different weight groups.

### Limitations

The cross-sectional design is a limitation of this study. A longitudinal study would have been more appropriate to detect causal relationships between obesity and healthcare-acquired infections and to compare the relative risks between the obese and the non-obese. The data are a reflection of the diversity of the hospitalized patient population in tertiary healthcare organizations. This study, however, provides baseline data for further research into the impact of obesity on antimicrobial dosing. In this study, we did not use acuity of illness scores. Acuity of illness may have been a confounder in this study.

### Conclusions

In this study, one in five hospitalized patients was obese and obesity was associated with more complicated antimicrobial therapy. Even though the evidence base is inadequate for some antimicrobials, no dosing adjustments were found to be made in any of the antimicrobial regimens of obese patients. Future studies need to investigate the effect of obesity on antimicrobial outcomes.

## Funding

This work is supported by: (i) the Royal Pharmaceutical Society Pharmacy Research and Practice Galen Award; (ii) the National Institute for Health Research Biomedical Research Centre Funding Scheme at Imperial College; (iii) the National Centre for Infection Prevention and Management (CIPM) funded by the United Kingdom Clinical Research Council (UKCRC G0800777); and (iv) the National Institute of Health Research Health Protection Research Unit, Healthcare Associated Infection and Antimicrobial Resistance at Imperial College London.

L. D. is supported in part by a National Institute of Health Research Career Development Award (NIHR CDF-2011-04-017) and the Cambridge Biomedical Research Centre. M. G., at the time of this research, was partly supported by the National Institute of Health Research Imperial Biomedical Research Centre (BRC).

## Transparency declarations

None to declare.

## Disclaimer

The views expressed are those of the authors and not necessarily those of the NHS, the NIHR, the Department of Health or Public Health England.
